# Temporal, Spatial and Seasonal Patterns of Parvovirus B19 Seroepidemiology in Childbearing-Aged Women in Croatia, 2015–2024

**DOI:** 10.3390/v17111477

**Published:** 2025-11-06

**Authors:** Tatjana Vilibić-Čavlek, Klara Barbić, Maja Bogdanić, Maja Mijač, Ana Sanković, Dan Navolan, Nadica Motofelea, Dana Liana Stoian, Sunčanica Ljubin-Sternak

**Affiliations:** 1 Department of Virology, Croatian Institute of Public Health, 10000 Zagreb, Croatia; maja.bogdanic@hzjz.hr; 2 School of Medicine, University of Zagreb, 10000 Zagreb, Croatia; maja.mijac@stampar.hr (M.M.); suncanica.ljubinsternak@stampar.hr (S.L.-S.); 3 Statistics Concentrator, Harvard University, Cambridge, MA 02139, USA; klarabarbic@college.harvard.edu; 4 Department of Molecular Microbiology, Andrija Štampar Teaching Institute of Public Health, 10000 Zagreb, Croatia; 5 Department of Microbiology, University of Applied Health Sciences, 10000 Zagreb, Croatia; asankovic@zvu.hr; 6 Department of Obstetrics and Gynecology, “Victor Babes” University of Medicine and Pharmacy, 300041 Timisoara, Romania; navolan@umft.ro (D.N.); nadica.motofelea@umft.ro (N.M.); 7 Department of Clinical Practical Skills, “Victor Babes” University of Medicine and Pharmacy, 300041 Timisoara, Romania; 8 Department of Endocrinology, “Victor Babes” University of Medicine and Pharmacy, 300041 Timisoara, Romania; stoian.dana@umft.ro

**Keywords:** parvovirus B19, seroprevalence, childbearing-aged women, Croatia

## Abstract

Parvovirus B19 (B19V) infection during pregnancy represents a significant clinical concern due to its potential impact on the fetus and pregnancy outcome. We analyzed temporal, spatial, and seasonal B19V seroepidemiology in childbearing-aged and pregnant women in Croatia over 10 years (2015–2024). A total of 976 women who underwent routine TORCH screening were included. The overall B19V IgG and IgM seroprevalence was 63.7 and 8.7%, respectively, with significant temporal differences (IgG 36.8–72.3%, IgM 1.3–18.4). Significant differences in the IgG seropositivity were observed among age groups, from 42.3% in the 16–20 group to 72.7% in the 36–40 group. Spatial analysis showed no significant differences in the IgG or IgM seroprevalence between regions (City of Zagreb/Northern Croatia, Pannonian and Adriatic Croatia) and settlements (urban, suburban/rural). Additionally, seropositivity did not differ significantly in relation to obstetric history. In a logistic regression model, age was a significant predictor for IgG seroprevalence, with each additional year of age associated with increased odds of IgG seropositivity. Year, region, and settlement type were not significant predictors, indicating no evidence of temporal trends, regional differences, or differences between urban and rural settings after adjusting for age. Year of sampling, age, and region were significant predictors for IgM positivity. Acute infections were most common from March to July (61.2%) and December (9.4%), with regional variations in seasonal prevalence patterns. Given that screening in pregnancy is not routinely recommended, the high susceptibility to B19V observed in Croatia supports targeted serologic testing in women with clinical symptoms, known exposure, or occupational risk.

## 1. Introduction

Parvovirus B19 (B19V; *erythroparvovirus primate 1* according to the new ICTV classification) is a small (18–26 nm), non-enveloped, single-stranded DNA virus of the family *Parvoviridae*, genus *Erythroparvovirus* [1]. There are three main genotypes (1–3). Genotypes 1 and 2 are most commonly found in Western countries, such as the United States and Europe, whereas genotype 3 mostly circulates in restricted areas in sub-Saharan Africa and South America [2]. The viral genome consists of two large open reading frames encoding nonstructural protein 1 (NS1) and two capsid proteins: a major structural VP2 protein (95%) and a minor structural VP1 protein (5%) [3].

The B19V virus shows tropism and replicates in erythroid progenitor cells in the bone marrow and fetal liver, which largely explains many of its clinical manifestations. The main receptor for the virus is the blood group P antigen, expressed on the surfaces of various cell types, including erythroblasts, megakaryocytes, endothelial cells, fetal myocytes, hepatocytes, and placental trophoblasts [4,5].

Although B19V infections occur globally, the prevalence varies by age and geographic location, generally being higher in developing countries and lower in more isolated communities. B19V infects only humans. In temperate climates, primary infections occur most commonly in school-aged children in the late winter, spring, and early summer, with periodic outbreaks occurring every three to six years [6]. B19V is primarily transmitted by the respiratory route, but can also be transmitted through blood transfusions, as well as vertically from a pregnant mother to her fetus [7]. Children aged 5–15 years are most frequently infected. Approximately 50% of children have antibodies by age 15, while 60–80% of individuals are seropositive by adulthood [5].

The “fifth disease” or *erythema infectiosum* is the most common clinical manifestation in children, characterized by a “slapped cheek” rash followed by rash on trunk and limbs. Arthritis and arthralgia of the small joints (hands, feet, and knees) may occur in older children and adults [8]. Patients with underlying conditions such as reduced red blood cell production (thalassemia) or increased red blood cell destruction (sickle cell anemia, hereditary spherocytosis), as well as individuals who have received allogeneic hematopoietic stem cell or solid organ transplants, and those who are HIV-positive, may develop an aplastic crisis. “Gloves and socks” syndrome typically affects young adults, although it can occur at any age [6].

The maternal-fetal transmission rate of B19V infection ranges from 17% to 33%, with the highest risk observed between 9 and 20 weeks of gestation [9]. During the first trimester of pregnancy, the P antigen is highly expressed on the trophoblastic cells of the chorionic villi. Its expression declines in the second trimester and becomes minimal in the placenta by the third trimester. On the fetal side, the P receptor is found on hematopoietic cells in the liver, which serves as the primary site of hematopoiesis between 9 and 24 weeks of gestation. In the second trimester, fetal red blood cell production is high, increasing more than 30-fold, making the fetus particularly susceptible to B19V infection. By the third trimester, the risk of infection declines significantly due to the shift of hematopoiesis from the liver to the bone marrow [10].

The potential effects of B19V infection during pregnancy include spontaneous abortion and non-immune fetal hydrops. The risk of spontaneous abortion is highest before 20 weeks of gestation, occurring in approximately 13% of cases before 20 weeks and decreasing to 0.5% thereafter. The likelihood of developing fetal hydrops depends on the gestational age at the time of infection: less than 5% in the first trimester, approximately 10% between 13 and 20 weeks, and less than 1% after 20 weeks [8].

Seroepidemiological studies conducted among childbearing-aged and pregnant women have shown variations in seroprevalence depending on the geographic region. There is little data on the prevalence of B19V in the Croatian population. In a seroepidemiological study conducted from 2010 to 2021, the seroprevalence in the general population was 64.1%, with significant differences between population groups, ranging from 42.5% in children and adolescents to 67.1% in the adult general population [11]. Higher seropositivity was found in patients who underwent liver, kidney, and pancreas transplantation in 2021 and 2022 (77.1%) [12]. The seroprevalence in pregnant women was 65.1% [11].

While B19V infection is usually asymptomatic or mild in healthy individuals, certain population groups are at risk of complications, including patients with hemolytic disorders (aplastic crises), immunocompromised patients (chronic infection with persistent anemia or bone marrow suppression), and pregnant women (fetal anemia, hydrops fetalis, or miscarriage) [6,8]. Given the importance of B19V as part of the TORCH profile, this study analyzed the temporal and spatial patterns of B19V seroepidemiology in childbearing-aged women in Croatia over 10 years.

## 2. Materials and Methods

### 2.1. Study Participants

The study included 976 childbearing-aged and pregnant women aged 16–45 years tested at the Croatian Institute of Public Health as part of the routine TORCH profile from January 2015 to December 2024. All participants of Croatian nationality were included. For this study, participants were classified by age (five-year age groups), geographic region, area of residence, and obstetric history. The seroprevalence was analyzed in two five-year periods (2015–2019 and 2020–2024) and yearly.

The median age of study participants ranged from 31 (IQR = 27–37) to 35 (IQR = 30–39) years, with no significant differences between the years (*p* = 0.127) (Figure S1).

According to the Nomenclature of Territorial Units for Statistics (NUTS), a hierarchical system used by the European Union, Croatian geographic regions were defined as the City of Zagreb/Northern Croatia (*n* = 582; 59.7%), Pannonian Croatia (*n* = 202; 20.7%), and Adriatic Croatia (*n* = 192; 19.6%) (Figure S2) [13].

Most participants resided in urban regions (*n* = 663; 67.9%), while 32.1% (*n* = 313) were from suburban/rural areas.

Data on obstetric history was available for 833 (85.3%) participants. Regarding the obstetric history, three groups were defined: non-pregnant women (*n* = 729; 87.5%), women with normal pregnancy (*n* = 68; 8.2%), and women with unfavorable obstetric history: previous spontaneous abortions, children with congenital malformations, and infertility (*n* = 36; 4.3%).

### 2.2. Serological Testing

Serological testing was performed using a commercial enzyme-linked immunosorbent assay that contains recombinant viral structural protein as the antigen (Anti-Parvovirus B19 ELISA IgM/IgG ELISA; Euroimmun, Lübeck, Germany). The results were interpreted according to the manufacturer’s recommendations, as follows: IgM ratio <0.8 negative; 8.8–1.1 borderline; >1.1 positive; IgG International Units (IU)/mL <4 negative; 4–5.5 borderline, and ≥5.5 positive. Samples with detected only IgM antibodies were retested after a two-week interval and interpreted as false positives if seroconversion was not documented. Furthermore, samples with initially borderline IgM/IgG results were retested, and those that remained borderline upon repeat testing were classified as negative.

### 2.3. Statistical Analysis

Descriptive statistics were used to summarize the analyzed variables (age, region, settlement, and diagnosis). Annual IgG/IgM seroprevalence was estimated with 95% CIs (Wald for figures; Wilson for tables). Group differences across predefined age groups were tested with Pearson’s Chi-squared test. Temporal patterns and predictors were assessed with binomial logistic regression (univariate: calendar year; multivariate: year, age, region, settlement), with 95% CIs for coefficients; model-based predicted probabilities were visualized as age by year heat maps, separated by three predefined regions. Seasonal trends are shown using stacked monthly counts and region–month prevalence heat maps. Complete case analyses were used. Figures were created using ggplot2 (panels with patchwork), tables with knitr/kableExtra. For two-sided tests, the test size was α = 0.05, and *p*-values < 0.05 were considered significant. Analyses were performed in R4.4.2 (R Foundation for Statistical Computing, Vienna, Austria).

## 3. Results

### 3.1. Temporal Patterns of Parvovirus B19 Seroprevalence

**Figure 1:**
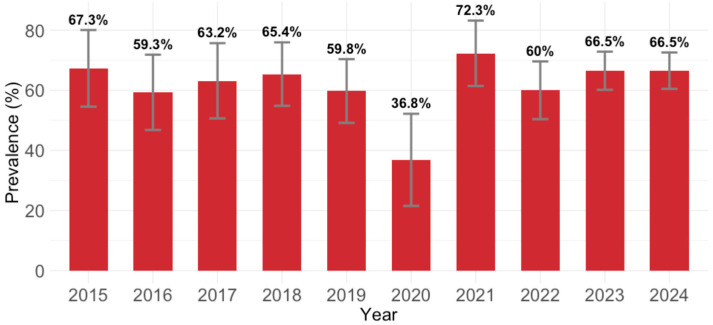
Yearly parvovirus B19 IgG seroprevalence (% with 95% confidence intervals).

B19V IgG antibodies were detected in 623/976 (63.7; 95% CI: 60.6–66.7) participants. Analyzing the yearly IgG seroprevalence, except for 2020 (36.8%; 95% CI = 21.8–54.0), the seropositivity varied from 59.3% (95% CI = 45.7–71.9; 2016) to 72.3% (95% CI = 59.8–82.7; 2021). These temporal differences were statistically significant (*p* = 0.040; Figure 1).

**Figure 2:**
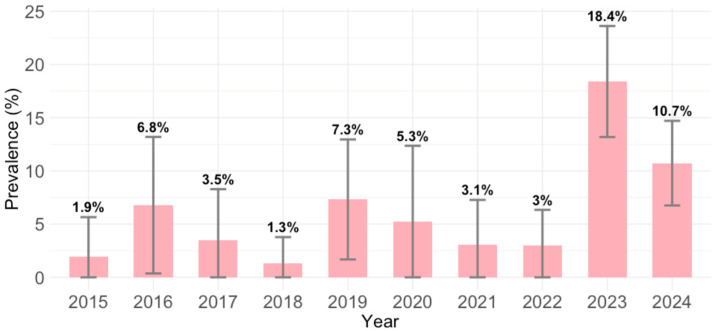
Yearly parvovirus B19 IgM seroprevalence (% with 95% confidence intervals).

B19V IgM antibodies were detected in 85/976 (8.7; 95% CI = 7.0–10.7) participants. Comparing the yearly seroprevalence rates, significant differences in seropositivity were observed between years (*p* < 0.001). The prevalence of acute infections was lowest in 2018 (1.3%; 95% CI = <0.1–6.9), and highest in 2023 (18.4%; 95% CI = 13.4–24.3) and 2024 (10.7%; 95% CI = 7.1–15.4) (Figure 2).

### 3.2. Age-Related Patterns of Parvovirus B19 Seroprevalence

Analyzing the seropositivity rate by age group, significant differences (*p* < 0.001) in the IgG seropositivity were observed between age groups. The lowest seropositivity was reported in the 16–20 age group (42.3%; 95% CI = 29.9–55.8), followed by a sharp increase in the 21–25 age group (68.0%; 95% CI = 58.2–76.5), with no obvious trend afterward (Table 1).

**Figure 3:**
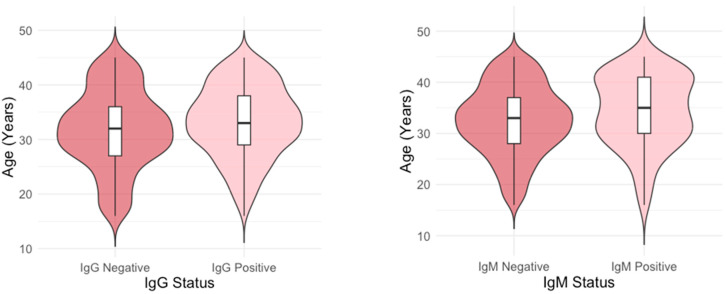
Age distribution of parvovirus B19 IgG and IgM seropositive and seronegative participants. The width of the violin represents the frequency of seropositive at a given age. A wider violin at older age in IgG seropositive individuals indicates that more positive individuals are in older age groups, and a shift in the median that older individuals are more likely to be seropositive. The violin in IgM seropositive individuals is bimodal (two peaks at 30.9 and 41.3 years, respectively).

The age distribution differed significantly between IgG-positive and IgG-negative individuals (*p* = 0.003), as well as between IgM-positive and IgM-negative individuals (*p* = 0.024). Median age of B19V IgG-positive and negative participants was 33 (IQR = 29–38 and 32 (IQR = 27–36) years, respectively, while median age of B19V IgM-positive and negative participants was 35 (IQR = 30–41) and 33 (IQR = 28–37) years (Figure 3).

A linear regression analysis showed that IgG seropositive individuals were, on average, 1.38 years older than those who were seronegative (β = 1.38, SE = 0.45, 95% CI = 0.493, 2.274, *p* = 0.002). In addition, IgM seropositive individuals were, on average, 1.73 years older than those who were seronegative (β = 1.73, SE = 0.78, 95% CI: [0.21, 3.26], *p* = 0.026).

### 3.3. Spatial Patterns of Parvovirus B19 Seroprevalence

Spatial analysis of the overall B19V seroprevalence showed no significant differences in the IgG (*p* = 0.084) and IgM (*p* = 0.060) seropositivity between regions. The highest IgG seroprevalence was found in Zagreb/Northern Croatia (385/582; 66.1%, 95% CI = 62.0–69.7), followed by Adriatic Croatia (122/192; 63.9%, 95% CI = 56.9–70.4) and Pannonian Croatia (116/202; 57.4%; 95% CI = 50.5–64.0) (Figure S3).

Acute B19V infections were more frequent in the Adriatic Croatia (25/192; 13.1%, 95% CI = 9.8–18.6) than in Pannonian Croatia (16/202; 7.9%, 95% CI = 4.9–12.5) and the City of Zagreb/Northern Croatia (44/582; 7.6%; 95% CI = 5.7–10.9) (Figure S4).

Analyzing the IgG prevalence by settlement type, no significant differences (*p* = 0.732) were found between residents of urban (425/663, 64.1%, 95% CI = 60.4–67.7) and suburban/rural areas (198/313, 63.3%; 95% CI = 57.8–68.4) (Figure S5).

Similarly, prevalence of acute infections did not differ significantly (*p* = 0.427) between urban and suburban/rural regions (61/663, 9.2%, 95% CI = 7.2–11.6; vs. 24/313, 7.7%, 95% CI = 5.2–11.2) (Figure S6).

### 3.4. Parvovirus B19 Seroprevalence by Obstetric History

The observed differences in the B19V IgG seroprevalence by obstetric history were not significant (*p* = 0.760): 469/729 (64.3%; 95% CI = 60.8–67.7) in non-pregnant women, 41/68 (60.3%, 95% CI = 48.4–71.1) in pregnant women with normal pregnancy, and 24/36 (66.7%; 95% CI = 50.3–79.8) in women with unfavorable obstetric history (Figure S7).

Like IgG seroprevalence, the IgM seroprevalence did not differ between the diagnosis groups (*p* = 0.122): non-pregnant women 73/729 (10.0%; 95% CI = 8.0–12.4), normal pregnancy 3/68 (4.4%; 95% CI = 1.5–12.1); unfavorable obstetric history 1/36 (2.8%; 95% CI = 0.5–14.2) (Figure S8).

### 3.5. Risk Analysis for Parvovirus B19 IgG and IgM Seropositivity

**Figure 4:**
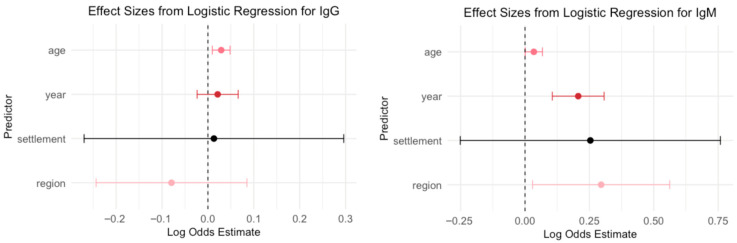
Logistic regression analysis for the risk of parvovirus B19 IgG and IgM seropositivity (log odds with 95% confidence intervals; CI). A coefficient >0 means the predictor is associated with increased odds of being positive, and a coefficient <0 means decreased odds of being positive. Predictors whose CI exclude 0 are statistically significant.

A logistic regression model was used to evaluate the association between demographic variables (year of sampling, age, region, and settlement type) and B19V seropositivity (Figure 4).

Age emerged as a significant predictor for IgG seroprevalence: each additional year of age was associated with increased odds of IgG seropositivity (OR = 1.03, *p* = 0.003), suggesting that older individuals were more likely to be IgG seropositive. Year, region, and settlement type were not statistically significant predictors in this model (*p* > 0.35 for all), indicating no clear evidence of temporal trends, regional differences, or differences between urban and rural settings after adjusting for age.

The year of sampling was a strong and significant predictor for IgM seroprevalence: each additional year was associated with higher odds of IgM seropositivity (OR = 1.23, *p* < 0.001), indicating a temporal increase in recent or acute infections over time. Age was also a significant predictor (OR = 1.03, *p* = 0.047), suggesting that older individuals had slightly higher odds of being IgM seropositive.

The region showed a modest but statistically significant association (OR = 1.34, *p* = 0.030), indicating regional differences in IgM prevalence. In contrast, settlement type (urban vs. rural/suburban) was not a significant predictor (*p* = 0.980), suggesting no meaningful difference in IgM seropositivity based on urbanization level in this adjusted model.

The association between settlement type, region, and obstetric history and IgG/IgM seropositivity, adjusting for year of sampling, was evaluated by a logistic regression model (Table 2 and Table 3).

There was no statistically significant association between settlement type and IgG seropositivity (urban vs. rural, *p* = 0.971). Among the calendar years, only 2020 showed a significant decrease in odds of IgG seropositivity compared to the reference year (2015; *p* = 0.004). No other year showed a statistically significant or borderline trend. This suggests a potential temporal dip in IgG prevalence, specifically in 2020. 

Compared to participants from the City of Zagreb/Northern Croatia, those from Pannonian Croatia had significantly lower odds of IgG seropositivity (OR = 0.69, *p* = 0.031). The difference between Adriatic Croatia and Zagreb/Northern Croatia was not statistically significant (*p* = 0.561). Among the sampling years, only 2020 showed a significant decline in the odds of IgG seropositivity compared to 2015 (*p* = 0.005). No other years demonstrated statistically significant associations. This suggests a potential regional disparity in exposure or immunity, and a notable drop in IgG positivity in 2020.

Analyzing the obstetric history, compared to participants who were not pregnant, neither group with normal pregnancies (*p* = 0.576) nor group with unfavorable obstetric history (*p* = 0.685) was significantly associated with odds of IgG seropositivity. Among the sampling years, only 2020 was significantly associated with lower odds of IgG seropositivity compared to 2015 (*p* = 0.004), indicating a potential temporal decline during that year. No other year showed a statistically significant association (Table 2).

The logistic regression model (Table 3) found no significant difference in IgM positivity between rural and urban participants (*p* = 0.983). Among the years included in the model, only 2023 was significantly associated with increased odds of IgM seropositivity compared to 2015 (*p* = 0.016). Year 2024 showed a borderline significant increase in IgM positivity (*p* = 0.072), while all other years did not show significant associations. This suggests a potential temporal increase in IgM seropositivity in 2023, with the trend possibly continuing into 2024.

After adjusting for calendar year, participants from Adriatic Croatia had significantly higher odds of IgM seropositivity compared to those from the City of Zagreb/Northern Croatia (OR =1.86, *p* = 0.019). There was no significant difference between Pannonian Croatia and Zagreb/Northern Croatia (*p* = 0.941).

Regarding the obstetric history, neither normal pregnancies (OR = 0.44, *p* = 0.185) nor pregnancies with previous unfavorable obstetric history (OR = 0.32, *p* = 0.276) were significantly associated with differences in IgM seropositivity compared to non-pregnant participants. Among the calendar years, only 2023 showed a significant increase in odds of IgM positivity (*p* = 0.023). No other year was significantly associated with changes in IgM seropositivity (Table 3).

**Figure 5:**
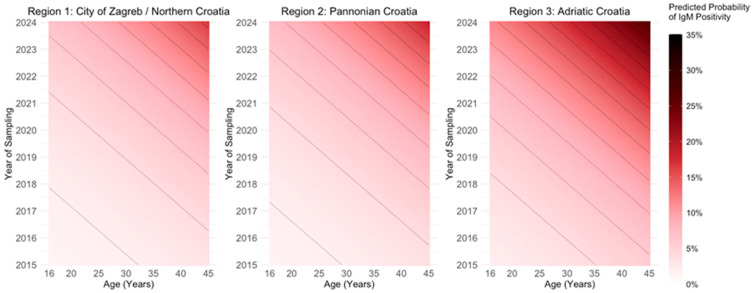
Significant predictors for parvovirus B19 IgM positivity from logistic regression. Each panel displays a heat map where the x-axis represents age, the y-axis represents the year of sampling, and the color intensity represents the predicted probability of IgM positivity (darker shades of red for higher probabilities). Contour lines on the graphs connect the points on the heat map (combinations of age and year of sampling) with the same predicted probability of IgM positivity. Contour lines that are closer together indicate a steeper increase in probability.

Figure 5 shows the predicted probability of IgM positivity based on age and year of sampling, plotted separately for each of the three Croatian regions: City of Zagreb/Northern Croatia, Pannonian Croatia, and Adriatic Croatia. All these estimates are based on a multivariate logistic regression model that includes all significant predictors (age, year, and region) for IgM positivity. The plot reveals a consistent trend across regions, with the probability of IgM detection increasing with both age and sampling year. This suggests that older individuals sampled in more recent years were more likely to test positive for IgM.

### 3.6. Seasonal Distribution of Parvovirus B19 Infections

**Figure 6:**
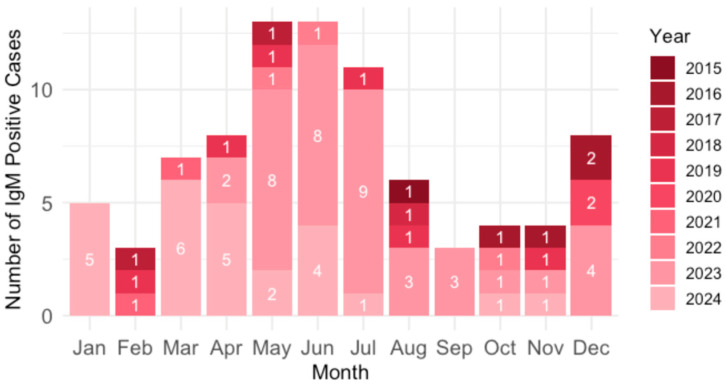
Seasonal distribution of parvovirus B19 infections by year.

Analysis of the B19V seasonality showed that acute infections were most common in spring and summer (March–July; 61.2%) and December (9.4%) (Figure 6).

**Figure 7:**
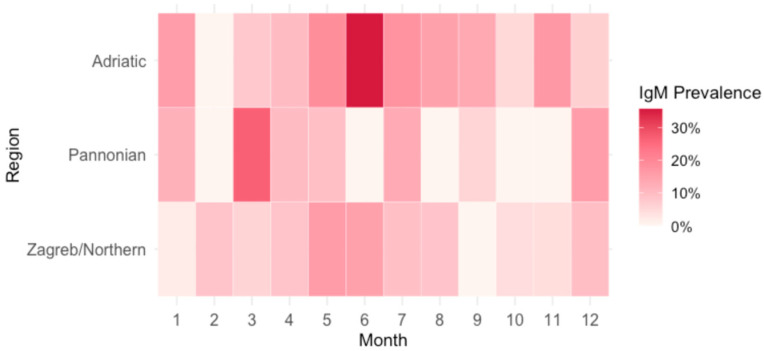
Parvovirus B19 IgM seroprevalence by region and month.

Figure 7 represents seasonal trends over all observed years in three different regions: City of Zagreb/Northern Croatia, Pannonian Croatia, and Adriatic Croatia. Regional variations in seasonal prevalence patterns were observed. Adriatic Croatia demonstrated a pronounced peak in June, whereas Pannonian Croatia exhibited an atypical peak in March. In contrast, the City of Zagreb/Northern Croatia displayed a more uniform distribution of cases throughout the year, with less pronounced month-to-month fluctuations compared to the other two regions. Additionally, the highest prevalence in the City of Zagreb/Northern Croatia (15.7%) was lower compared to the other two regions (Pannonian 26.7%, Adriatic 35.7%). City of Zagreb/Northern Croatia and Adriatic Croatia showed characteristic seasonal trends for B19V infections.

## 4. Discussion

The overall B19V IgG seroprevalence of 63.7% in the Croatian childbearing-aged and pregnant women detected in this study is similar to the seroprevalence in Denmark (58.2%) [14] and Norway (57.4–63.1%) [15]. In addition, the proportion of the Croatian seronegative women is similar to the estimates of the average proportion of susceptible childbearing-aged women in England and Wales (38.1%), Italy (39.9%), and Poland (36.8%) [16]. Lower seropositivity rates were found in some other European countries: 37.8 to 40.4% in Bulgaria [17,18], 43.6% in Poland [19], and 51.12% in Serbia [20], while higher seropositivity was found in the Netherlands (70.0%) [21]. In Germany, the highest seropositivity was observed among women with two or more children (81.6%) and those working with children under 6 years of age (88.9%). In contrast, significantly lower seroprevalence was found in age-matched single women (64.8%) and in women whose work involved contact with children over 6 years and adolescents (63.8%) [22].

Analyzing the seropositivity in Croatia by calendar year, except for 2020 (B19V IgG seroprevalence 36.8%), the seropositivity rates varied between years, ranging from 59.3% (2016) to 72.3% (2021). The emergence of SARS-CoV-2, causing the COVID-19 pandemic, could be the possible reason for the low overall seroprevalence in 2020. During the pandemic, fewer routine screenings were performed. Healthcare systems were overwhelmed, and non-COVID testing was often deprioritized. In 2020, the lowest number of participants were tested, which could at least in part contribute to the underestimation of the seroprevalence. Many schools were either closed or transitioned to remote instruction, leading to a marked reduction in social interactions among children. Decreased exposure in children, as the main virus reservoirs, especially those in school or daycare (common sources of B19V transmission), may lead to reduced viral circulation, causing a low incidence of B19V infection.

Studies conducted in the general population usually showed an age-related increase in B19V seroprevalence. However, among women within the childbearing age range (e.g., 16–45 years), some studies suggest that seroprevalence may not differ significantly across age groups. In our study, seroprevalence varied significantly across age groups, with the lowest seropositivity observed in individuals aged 16–20 years (42.3%), followed by a sharp increase in the 21–25-year age group (68.0%), and irregular, but relatively stable rates in subsequent age groups (ranging from 57.3% to 72.7%). The observed non-linear increase in IgG seropositivity with age suggests that exposure to B19V does not occur at a constant rate throughout life. After the peak exposure periods in childhood and early adulthood, when social and occupational contact rates are higher, the rate of new infections slows, resulting in a plateau or irregular pattern of seropositivity among older age groups. In addition, the population-specific exposure pattern, such as differences in childcare contact, occupation, or living conditions, may contribute to deviations from a strictly linear trend. However, age emerged as a significant predictor for B19V IgG seroprevalence in the logistic regression model, with each additional year of age associated with increased odds of IgG seropositivity. Similar age-related increase in seroprevalence was observed in Bulgaria [17], Serbia [20], and Germany [22].

A marked rise in seroprevalence after the age of 20 may be attributed to increased social interaction. People in their early twenties often begin attending college and workplaces, which increases their exposure to others and, consequently, to the virus. Shared living environments, such as dormitories and apartments, further facilitate transmission. Additionally, some young adults start working in childcare, healthcare, or education settings where contact with infected children, as the major virus reservoir, increases the risk of infection. Higher seropositivity in older age groups reflects the cumulative exposure to the virus over time. Once infected individuals develop long-lasting antibodies. In addition, some individuals might be repeatedly exposed throughout life, boosting their immune response and maintaining detectable antibody levels.

No significant differences were detected between age groups in Denmark [14]. Moreover, no significant difference in seropositivity was observed between pregnant women and non-pregnant women (general population) of the same age in Taiwan [23]. As B19V infection is typically acquired during childhood, a substantial proportion of women are already seropositive by childbearing age, resulting in a plateau in seroprevalence.

Regarding the settlement type, as in the previous Croatian study [11], this study found no significant difference in the seroprevalence between residents of urban and suburban/rural areas (64.1 and 63.3%, respectively). Like our results, no association between the residence type and B19V seroprevalence was reported in Poland [24] and Bulgaria [17].

In Iran, a higher seropositivity rate was observed among urban residents compared to those in rural areas (88% vs. 84.3%) [25]. Urban areas have higher population density. Since B19V is primarily transmitted by respiratory droplets, crowded environments such as schools, public transport, and workplaces may increase the risk of transmission. Children in daycare centers, schools, and playgrounds, who are common B19V reservoirs, are more densely concentrated in urban environments. Additionally, better surveillance and diagnostic capabilities may also contribute to the higher seroprevalence. Urban areas often have better access to healthcare and laboratory testing, leading to higher rates of case detection and reporting.

In contrast, in Germany, residents of smaller cities showed a higher seropositivity rate (74.8%) than those living in larger cities (69.0%) [22]. In smaller cities, children are likely to have closer community contacts, which can lead to earlier exposure in life. Conversely, larger cities often benefit from better infrastructure, potentially reducing the risk of transmission in settings like schools and households. Limited access to healthcare, sanitation, or health education in smaller cities may also contribute to higher rates of infection.

In the present study, neither the group with an unfavorable obstetric history nor the group with normal pregnancies was significantly associated with the odds of IgG seropositivity compared to non-pregnant participants. Similarly, in a population-based pregnancy cohort study conducted in Norway, no significant differences were observed in the IgG seropositivity in the case group (women with late miscarriage and perinatal death) and controls with live-born children (57.4 and 63.1%, respectively) [15].

Analyzing yearly differences in the IgM seropositivity in our study, higher IgM rates in certain years (2023, 2024) indicated B19V outbreaks. Global cyclical epidemiology patterns suggest that B19V infections tend to have epidemic peaks every 3–5 years [7]. The COVID-19 pandemic disrupted this pattern, resulting in unusually low circulation from around 2020–2022. Widespread mask use, social distancing, school closures, improved hygiene, and reduced travel significantly decreased transmission of many respiratory and droplet-borne viruses, and possibly B19V.

Although the B19V is typically associated with childhood infections, it can infect individuals at any age. The prevalence of acute infections in participants tested in this study showed an interesting demographic shift from the expected trend. In all three tested regions, older individuals sampled in more recent years were more likely to test IgM positive. The occupational or parental exposure risk can explain this observation. Adults aged 35–45 are more likely to have young children at home or work in settings such as schools or healthcare facilities, where exposure to infected children is more common. A recent outbreak (2023–2024) may have coincided with increased susceptibility or heightened exposure within this age group. In addition, adults are more likely to seek medical attention when symptoms appear, increasing the likelihood of being tested and diagnosed. The increased probability of IgM detection observed in the most recent years (2023 and 2024) is at least partially attributable to the outbreak that occurred during that period.

Like the B19V epidemic in 2023–2024 in Croatia, several European Union and European Economic Area (EU/EEA) countries reported substantial increases in the detection of B19V during late 2023 and early 2024. Increased B19V circulation has been recorded in the Czech Republic, Denmark, France, Ireland, Latvia, Lithuania, the Netherlands, Norway, and Spain [26]. In Denmark, laboratory-confirmed cases of B19V infection surged in early 2024, with peak incidence about 3.5 times higher than during the previous epidemic in 2017 [27]. Furthermore, a study from France reported similar increasing trends of B19V detection [28]. A notable increase in B19V circulation was also observed among pregnant women in Italy, starting in late 2023 [10].

Analyzing the seasonal distribution of acute B19V infections, similar to the European epidemiological patterns (late winter, spring, and early summer) [26], most B19V infections in the 2023–2024 Croatian outbreak were recorded from March to July (61.2%) and December (9.4%). Regional differences in the seasonal distribution of acute infections may be attributed to a combination of factors. Climatic variations likely play a significant role in shaping seasonal transmission patterns. Adriatic Croatia, characterized by a Mediterranean climate with warmer and drier summers, exhibited a peak prevalence in June. This aligns with established seasonal trends for B19V, which typically show increased transmission during late spring and early summer, potentially due to increased outdoor social interaction and school-related exposure. In contrast, Pannonian Croatia demonstrated a peak in March, which may reflect the impact of colder continental climate conditions that promote indoor crowding during winter and early spring, facilitating respiratory virus transmission. Differences in population dynamics and mobility may also contribute to regional variation. Adriatic Croatia experiences significant seasonal population increases due to tourism during the summer months, which may amplify transmission during that period.

There are some limitations of the study that need to be addressed. Although IgM antibodies are usually considered a marker of acute infection, detection of B19V IgM antibodies may occasionally yield false-positive results due to nonspecific reactivity or cross-reactivity with antibodies produced during infections caused by other herpes group viruses, such as Epstein–Barr virus, cytomegalovirus, or herpes simplex virus [29,30]. Additionally, IgM antibodies may persist for several months following acute infection, which can complicate the interpretation of serological results. Confirmation by PCR for viral DNA or by monitoring IgG seroconversion in paired serum samples is recommended in these cases [31,32], which was not available in this study. Therefore, higher IgM prevalence reported in older individuals may be a result of background antibody reactivity or cross-reactivity due to exposure to other viruses.

## 5. Conclusions

The results of this study showed that almost 40% of Croatian childbearing-aged women are B19V seronegative and therefore susceptible to primary infection during pregnancy. The obtained results have several important implications for clinical practice and public health. Given the potential risks to the fetus, these results highlight the need for increased awareness, surveillance, and consideration of targeted screening strategies, especially during outbreaks or for women in high-risk environments like healthcare or childcare settings. While routine screening for B19V in pregnancy is not standard practice, the notable susceptibility rate identified in this study supports implementing targeted serologic testing in women with rash illness, known exposure, or occupational risk. Since no association was observed between obstetric history and IgG seropositivity, the seronegativity to B19V as a potential concerning indicator needs to be demonstrated. In addition, the possible correlation between B19V seropositivity and internal organ damage or clinical manifestations of active infection should be further investigated.

## Figures and Tables

**Table 1:** Parvovirus B19 IgG and IgM seropositivity by age.

Age Group	Tested	Parvovirus B19 IgG		*p*	Parvovirus B19 IgM		*p*
	*n* (%)	*n* (%)	95% CI		*n* (%)	95% CI	
16–20 years	52 (5.3)	22 (42.3)	29.9–55.8	<0.001	3 (5.8)	2.0–15.6	0.052
21–25 years	97 (10.0)	66 (68.0)	58.2–76.5		5 (5.2)	5.4–13.0	
26–30 years	214 (21.9)	122 (57.3)	50.6–63.7		18 (8.5)	5.4–13.0	
31–35 years	275 (28.2)	176 (64.0)	58.2–69.4		20 (7.3)	4.8–11.0	
36–40 years	187 (19.2)	136 (72.7)	65.5–78.2		16 (8.5)	5.3–13.4	
41–45 years	151 (15.4)	101 (66.9)	59.0–73.9		23 (15.2)	10.4–21.8	
Total	976 (100)	623 (63.7)	60.6–66.7		85 (8.7)	7.0–10.6	

**Table 2:** Estimated risk for parvovirus B19 IgG seropositivity.

Year	Settlement IgG Odds Estimate	*p*	Geographic Region IgG Odds Estimate	*p*	Obstetric History IgG Odds Estimate	*p*
2015	Ref.		Ref.		Ref.	
2016	−0.345	0.384	−0.319	0.423	−0.057	0.894
2017	−0.181	0.653	−0.184	0.649	0.041	0.923
2018	−0.084	0.823	−0.102	0.787	0.203	0.623
2019	−0.324	0.382	−0.372	0.317	−0.075	0.852
2020	−1.259	0.004 *	−1.256	0.005 *	−0.976	0.047 *
2021	0.234	0.562	0.230	0.570	0.454	0.291
2022	−0.316	0.378	−0.323	0.371	−0.199	0.592
2023	−0.034	0.915	−0.018	0.954	0.141	0.682
2024	−0.035	0.913	−0.053	0.871	0.099	0.769

* Statistical significance.

**Table 3:** Estimated risk for parvovirus B19 IgM seropositivity.

Year	Settlement IgM Odds Estimate	*p*	Geographic Region IgM Odds Estimate	*p*	Obstetric History IgM Odds Estimate	*p*
2015	Ref.		Ref.		Ref.	
2016	1.307	0.249	1.228	0.280	1.155	0.325
2017	0.630	0.611	0.632	0.610	0.614	0.621
2018	−0.401	0.777	−0.437	0.758	−0.328	0.818
2019	1.409	0.198	1.385	0.206	1.341	0.229
2020	1.054	0.396	1.046	0.400	1.175	0.347
2021	0.464	0.708	0.410	0.740	0.421	0.734
2022	0.456	0.696	0.328	0.778	0.328	0.779
2023	2.451	0.016 *	2.395	0.019 *	2.337	0.023 *
2024	1.857	0.072	1.793	0.082	1.514	0.144

* Statistical significance.

## Data Availability

The original contributions presented in the study are included in the article; further inquiries can be directed to the corresponding author.

## Supplementary Materials

The following supporting information can be downloaded at: https://www.mdpi.com/article/10.3390/v17111477/s1, Figure S1: Age distribution of study participants by year; Figure S2: Geographic distribution of study participants; Figure S3: Spatial parvovirus B19 IgG seroprevalence (% with 95% confidence intervals); Figure S4: Spatial parvovirus B19 IgM seroprevalence (% with 95% confidence intervals); Figure S5: Parvovirus B19 IgG seroprevalence by settlement (% with 95% confidence intervals); Figure S6: Parvovirus B19 IgM seroprevalence by settlement (% with 95% confidence intervals); Figure S7: Parvovirus B19 IgG seroprevalence by obstetric history (% with 95% confidence intervals); Figure S8: Parvovirus B19 IgM seroprevalence by obstetric history (% with 95% confidence intervals).

## Abbreviations

The following abbreviations are used in this manuscript:

B19V	Parvovirus B19
NUTS	Nomenclature of Territorial Units for Statistics
CI	Confidence interval
OR	Odds ratio
