# Supplementary figures and images for: Temporal, Spatial and Seasonal Patterns of Parvovirus B19 Seroepidemiology in Childbearing-Aged Women in Croatia, 2015–2024
Source: Viruses. 2025 Nov 6;17(11):1477. doi: 10.3390/v17111477 (PMC12656865; doi:10.3390/v17111477)

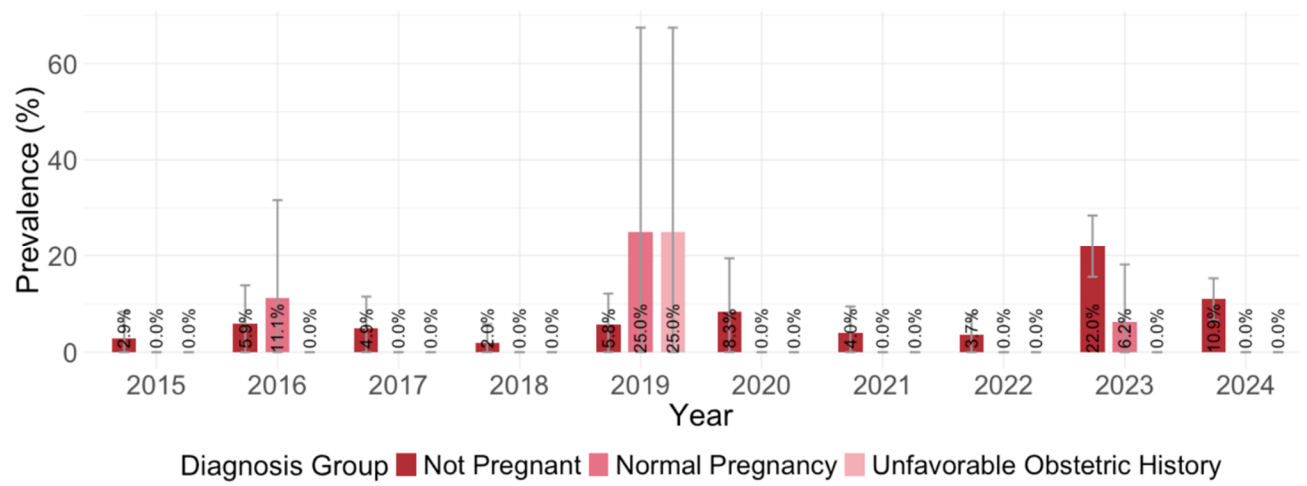

**Figure S8.** Parvovirus B19 IgM seroprevalence by obstetric history (% with 95% confidence intervals).

Supplement: Supplementary file 1 [file viruses-17-01477-s001.zip › Figure S8.pdf]

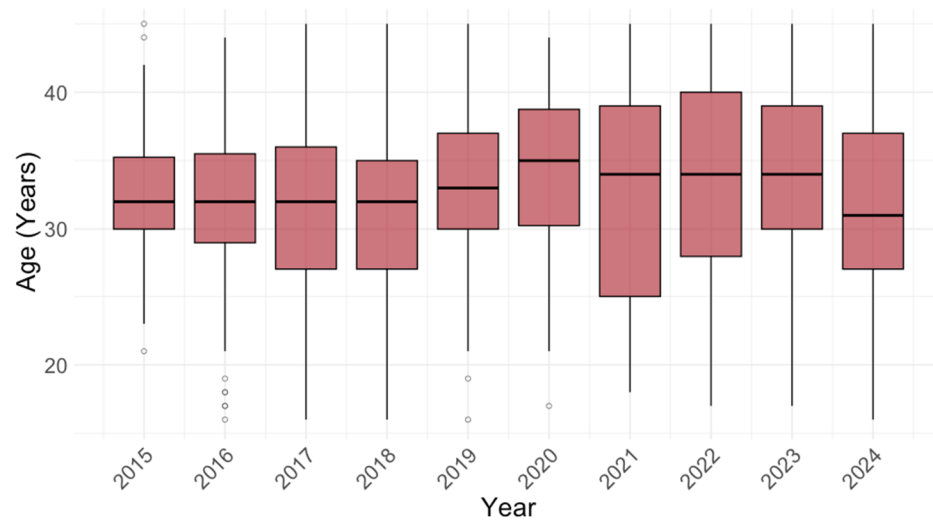

**Figure S1.** Age distribution of study participants by year.

Supplement: Supplementary file 1 [file viruses-17-01477-s001.zip › Figure S1.pdf]

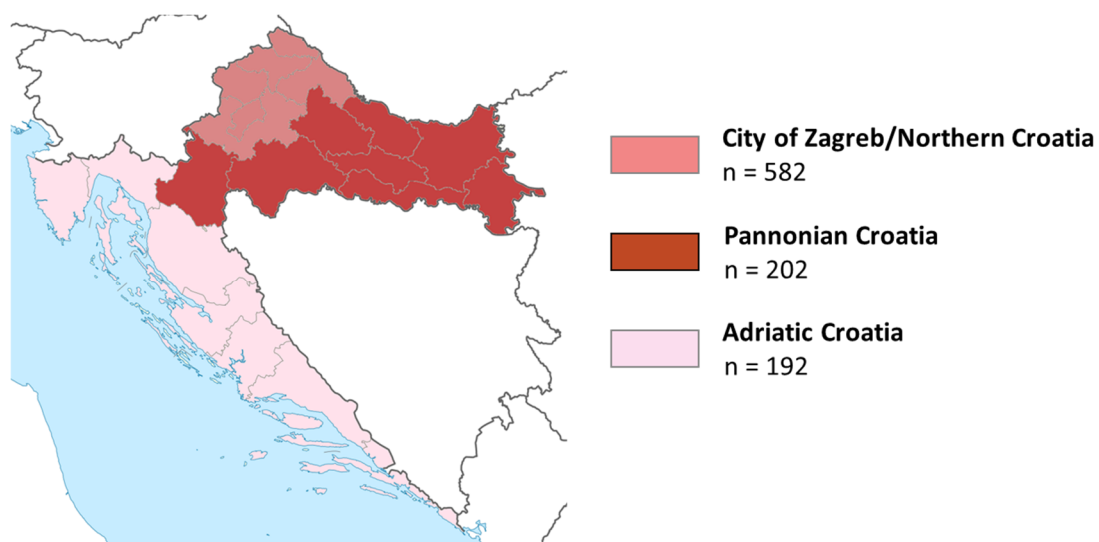

**Figure S2.** Geographic distribution of study participants.

Supplement: Supplementary file 1 [file viruses-17-01477-s001.zip › Figure S2.pdf]

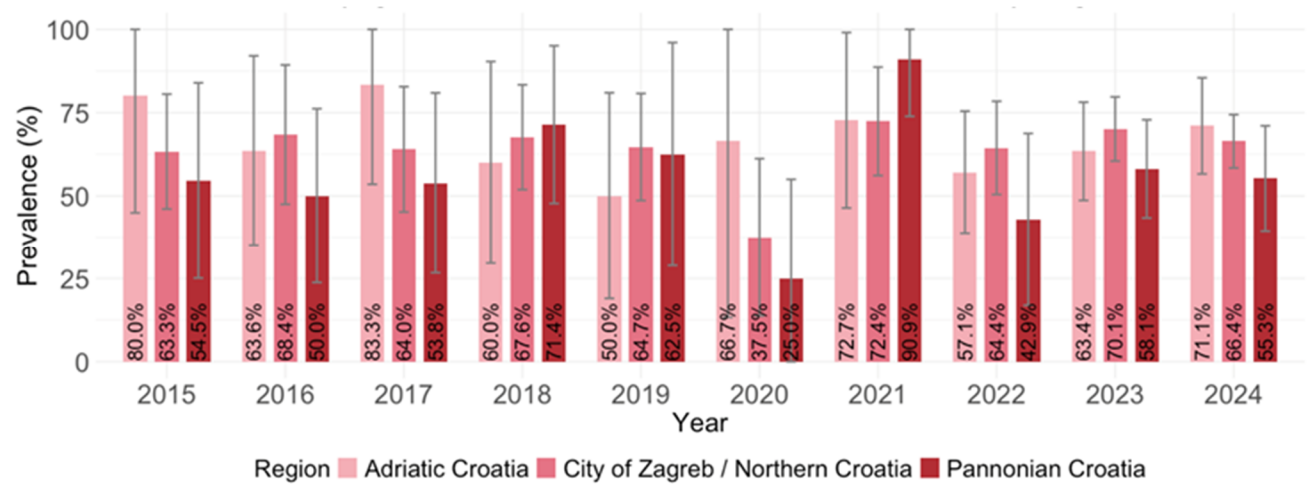

**Figure S3.** Spatial parvovirus B19 IgG seroprevalence (% with 95% confidence intervals).

Supplement: Supplementary file 1 [file viruses-17-01477-s001.zip › Figure S3.pdf]

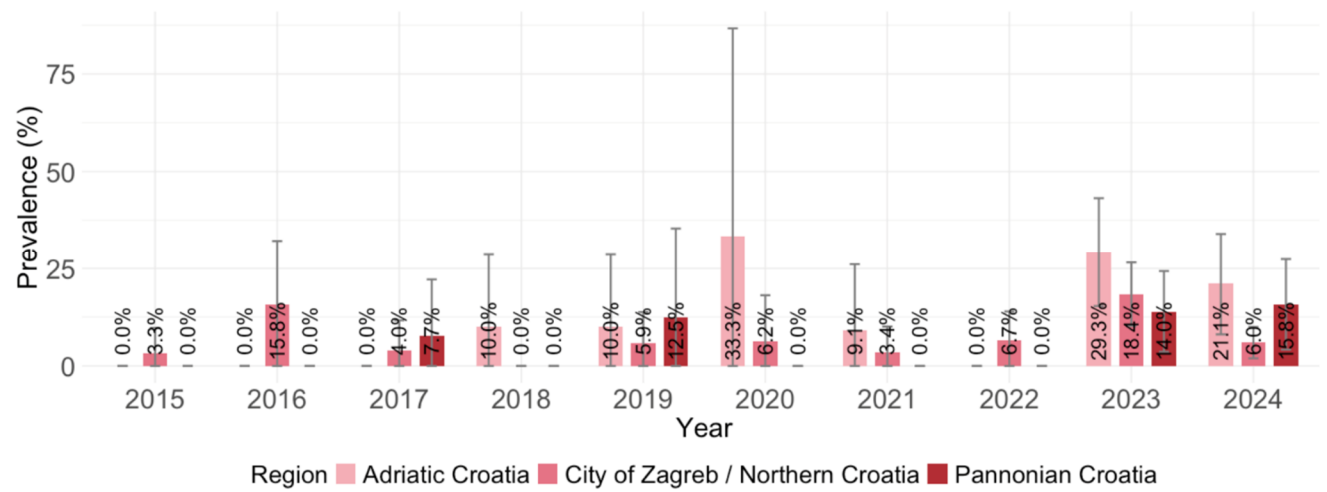

**Figure S4.** Spatial parvovirus B19 IgM seroprevalence (% with 95% confidence intervals).

Supplement: Supplementary file 1 [file viruses-17-01477-s001.zip › Figure S4.pdf]

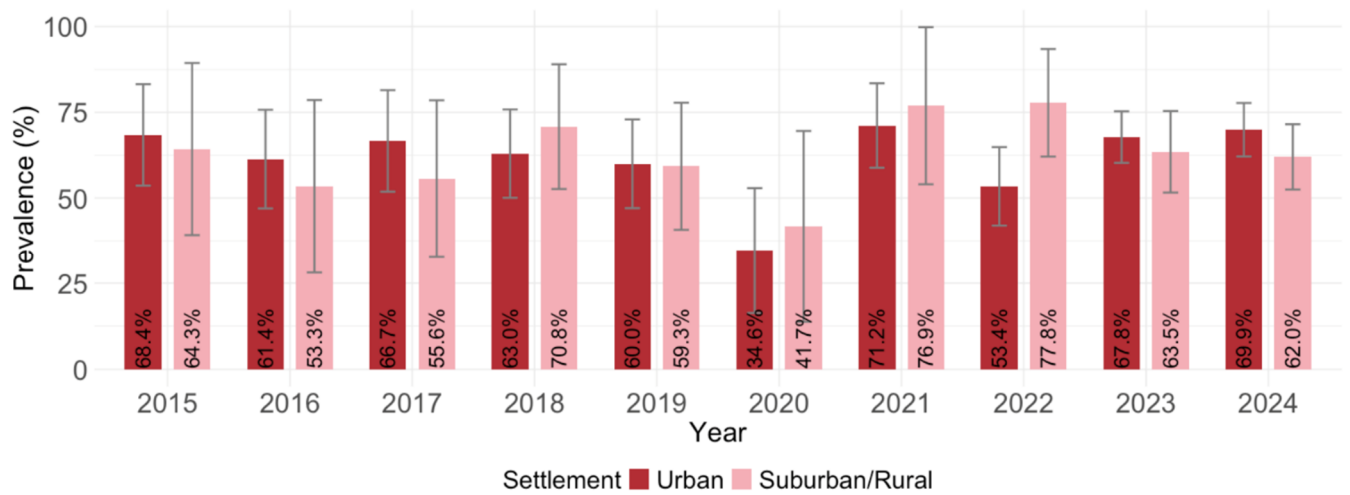

**Figure S5.** Parvovirus B19 IgG seroprevalence by settlement (% with 95% confidence intervals).

Supplement: Supplementary file 1 [file viruses-17-01477-s001.zip › Figure S5.pdf]

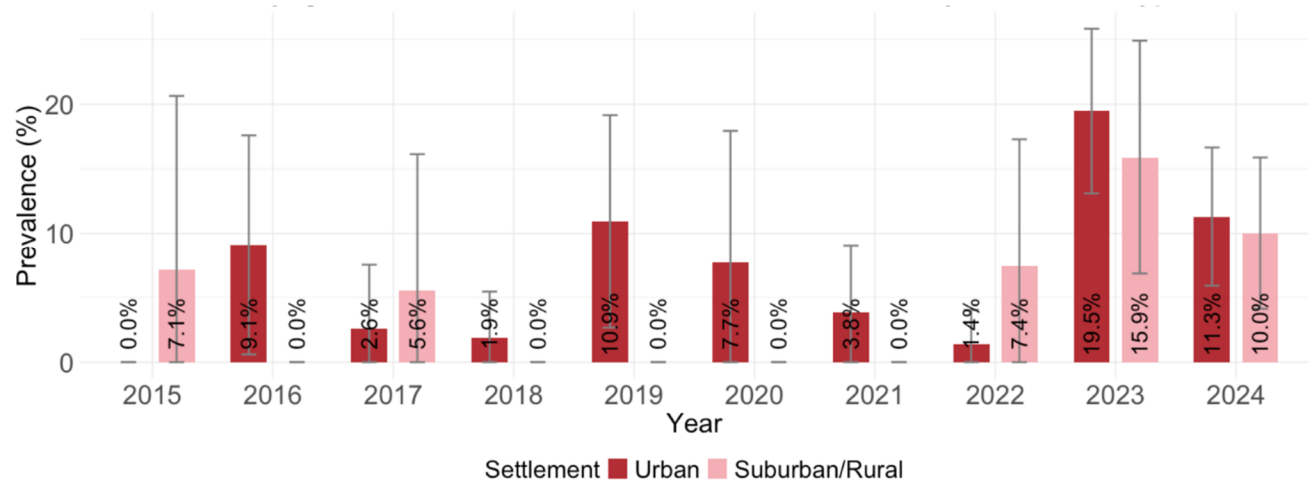

**Figure S6.** Parvovirus B19 IgM seroprevalence by settlement (% with 95% confidence intervals).

Supplement: Supplementary file 1 [file viruses-17-01477-s001.zip › Figure S6.pdf]

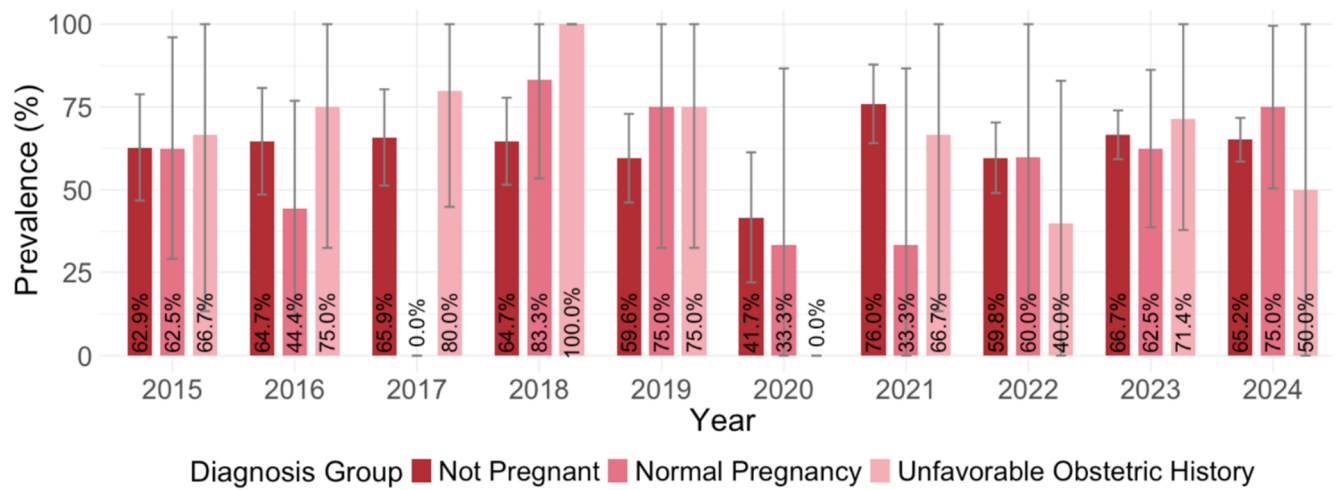

**Figure S7.** Parvovirus B19 IgG seroprevalence by obstetric history (% with 95% confidence intervals).

Supplement: Supplementary file 1 [file viruses-17-01477-s001.zip › Figure S7.pdf]
